# Personalized Consent Flow in Contemporary Data Sharing for Medical Research: A Viewpoint

**DOI:** 10.1155/2017/7147212

**Published:** 2017-05-30

**Authors:** Ester A. Rake, Marleen M. H. J. van Gelder, David C. Grim, Barend Heeren, Lucien J. L. P. G. Engelen, Tom H. van de Belt

**Affiliations:** ^1^Radboud REshape Innovation Center, Radboud University Medical Center, Nijmegen, Netherlands; ^2^Department for Health Evidence, Radboud Institute for Health Sciences, Radboud University Medical Center, Nijmegen, Netherlands

## Abstract

**Background:**

Health data personally collected by individuals with wearable devices and smartphones is becoming an important data source for healthcare, but also for medical research.

**Objective:**

To describe a new consent model that allows people to control their personally collected health data and determine to what extent they want to share these for research purposes.

**Methods:**

We developed, in close collaboration with patients, researchers, healthcare professionals, privacy experts, and an accredited Medical Ethical Review Committee, an innovative concept called “personalized consent flow” within a research platform connected to a personal health record. The development was an iterative process with informal meetings, semistructured interviews, and surveys. The final concept of the personalized consent flow was reviewed by patients and improved and approved by the same patients in a focus group.

**Results:**

This concept could result in optimal control for individual users, since they will answer questions about how they will share data. Furthermore, it enables users to collect data for specific studies and add expiration dates to their data. This work facilitates further discussion about dynamic and personalized consent. A pilot study with the personalized consent model is currently being carried out.

## 1. Introduction

A growing number of individuals use smartphones, tablets, and other wearable devices to measure health parameters, symptoms, and lifestyle factors. This development will influence healthcare and the way medical research will be conducted. Particularly, advanced sensing devices in combination with mobile Internet and cloud storage produce a continuous stream of data, probably resulting in earlier detection of clinical deterioration or recovery and improved performance of prediction models. Although the possibilities of using these data for medical research are promising, privacy and security issues remain challenging [1], especially for wearable devices [2]. Furthermore, there is a need for new consent models since consumers will be in control of their data, instead of researchers or physicians.

## 2. Self-Collected Data for Health Research

The potential benefits of using self-collected health data for healthcare and research in particular seem widespread. Last year, Apple introduced “ResearchKit,” an online tool to collect health data for medical research. It showed that efficient patient recruitment for medical studies in a short timeframe is possible: in a few hours 7,000 participants were recruited for a Parkinson study whereas the largest comparable study consisted of 1,700 subjects and recruitment took more than one year. Another example is PatientsLikeMe: an online platform on which 300,000 patients track their health and connect with other people having the same disease. Moreover, PatientsLikeMe users can donate their data for research and PatientsLikeMe launched the platform “Open Research Exchange” aiming to create health outcome measurements.

Although people seem willing to share their (self-collected) health data for the “general good” in order to contribute to research and new insights [3, 4], consenting only* once* for several (future) research purposes, which is also current practice for PatientsLikeMe and studies using ResearchKit, is not putting consumers in control of their data. Therefore, dynamic consent models comparable to those for biobank projects could be an acceptable solution to deal with this issue [5]. This allows consumers to share data for specific research projects and for a specific period of time. Although consumers can easily change their preferences, the data collection is still static: it does not take into account continuous data collection and donation by one subject. Meta consent models have been proposed for use of secondary health data [6], but these do not meet contemporary characteristics of continuously collected health data and data controlled in personal health records (PHR) either. In the UK, the care.data program is currently being implemented, which enhances disclosure of medical data for research by an opt-out system. Due to scarce communication and explanation, this opt-out system is still part of an on-going debate, with concerns about the private sector having access to merged health data [7]. These three examples show that it is challenging to implement a consent system that covers all aspects related to using self-collected health data for medical research.

## 3. Personalized Consent Flow

Therefore, we propose an innovative concept called “personalized consent flow” together with a research platform connected to a PHR to meet all characteristics of contemporary data collection and data sharing. Using this platform named “Here is my data” (HIMD), researchers can collaborate with users. This ensures that users have full control of their data and are able to share these for scientific research. This concept was developed in close collaboration with patients, the patient advisory board from a large academic hospital, the Dutch Patient Federation, researchers, healthcare professionals, privacy experts, and an accredited Medical Ethical Review Committee. It was an iterative process with informal meetings, semistructured interviews, and surveys. First, we performed a gap-fit analysis to structure the problem and identify the gaps between current methods of research with traditionally collected data compared to using self-collected health data with HIMD. A gap-fit analysis is a technique used to determine what steps are needed to move from a current state to a desired future state [8]. The gaps were identified based on analysis of relevant literature, regulations, laws, and reports. Second, semistructured interviews with incorporated stakeholders were conducted to obtain information about their opinions, needs, and stakes of the incorporated stakeholders. An interview guide, based on the human, organization, and technology-FIT framework was used [9]. The following themes were discussed: human factors, organizational factors, and technology factors, in relation to HIMD as a research platform. Furthermore, additional themes that emerged during the interview were also further discussed. The interviews were transcribed and overarching themes were identified by one author (Ester A. Rake) and discussed with a second author (Tom H. van de Belt) with expertise in qualitative research. These two information sources lead to identification of the fits and gaps related to ethics and regulations and the current situation regarding medical ethical research committees, consent, and research compared to the future situation using the research platform of HIMD. Third, we discussed hypothetical research casus using HIMD-data and possible consent choices for HIMD users with the Medical Ethical Review Committee, to learn more about the acceptability of different options. Moreover, options for future use of HIMD-data for research were identified and discussed with relevant stakeholders. This resulted in several concept versions of the personalized consent flow, of which the final version was reviewed by patients and improved and approved by the same patients in a focus group. Moreover, the Medical Ethical Review Committee also reviewed and approved the draft version of the personalized consent flow.

**Figure 1:**
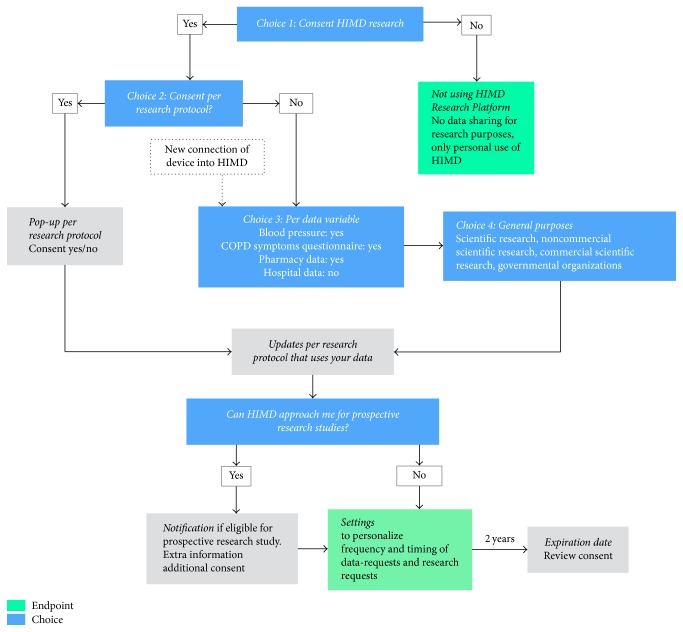
Outline of the personalized consent flow.

In this collaboration we determined that the consent flow should be personalized, transparent, and very simple. This could result in optimal control for individual PHR users and may increase trust among users [10]. Therefore, three main features characterize the proposed consent flow (Figure 1). First, users are asked general questions about sharing data. When they wish to share data for scientific research, they may opt for “narrow” consent, which implies that their consent will be asked for every individual study for which their data is of interest, or “broad” consent, which can be used for multiple studies. Furthermore, users are able to decide which data will be shared for specific studies and with whom. Logically, users receive similar questions when they add new data sources to their PHR, such as wearable devices. This principle is similar to changing privacy settings on Facebook. Second, users can choose to share existing data that they have collected passively, to share prospectively, collect data, or both. For prospective studies, researchers can invite specific users to collect selected data during a specific time period (e.g., heart rate with a wearable for the next two weeks), which is called a “research request” for active data collection. This is possible since the system allows them to identify potential study participants anonymously, based on their consent choices. Users can also be notified about future studies by signing up for the research program, which was already proposed by Mandl and Kohane in 2008 [11]. Third, expiration dates are connected to each consent choice, which ensures that a user reconsiders his decision. A default expiration date of one year will be assigned, but users may also select personal expiration dates, such as an expiration date connected to the duration of the study. Obviously, users can quit sharing data at any time. During all steps, users are informed about implications of consent options by using innovative consent tools such as videos, information graphics, and appealing examples. This ensures that users know what they consented for, in contrast with many existing platforms that have lengthy terms and conditions that discourage users from reading, who subsequently sign an uninformed consent.

Although we feel that the personalized consent flow would result in a novel participatory research design in which self-collected data is efficiently used and shared, some challenges exist. First, it is challenging to create the technical design that meets all necessary characteristics. Second, the design should efficiently deal with inactive users. Finally, user empowerment potentially jeopardizes the creation of optimal data sets since users can quit whenever they want. Therefore, an optimal balance between user empowerment and commitment has to be found.

## 4. Conclusion

Personally collected health data will become an important data source for medical research. New consent models are urgently needed to allow people to control their personally collected health data and determine to what extent they want to share these for research purposes. The personalized consent flow proposed could serve as a starting point to meet all requirements for sharing personally collected and controlled health data for research. This work facilitates further discussion about dynamic and personalized consent. A pilot study with the personalized consent model is currently being carried out.
